# Glances at the Hospitals

**Published:** 1904-10-08

**Authors:** 


### GLANCES AT THE HOSPITALS.

### LANCASTER ROYAL INFIRMARY.

At the beginning of the year there was a balance due to the treasurer of £438, and at the end of the year it had been reduced to £226. The total income was £3,486, and we note that the workpeople's own subscriptions and donations amounted to £715. The committee have also been fortunate in obtaining several donations during the year. Miss Woods sent £100, Mr. Joseph Torbock £50, and there were two anonymous gifts: one a Finsen Lamp and the other a complete modern operation table. The endowment fund has also been increased by £390. The cost per bed occupied is not given, nor the cost per head per week, and we did not notice any statement relative to the average duration of treatment. The infirmary contained 45 patients on January 1st, and 576 were admitted during the year, making a total of 621 under treatment. Among this number were 27 private patients, and these paid to the infirmary a total sum of £262. The medical and surgical tables show the numbers "recovered," "relieved," "not relieved," and "died." There were 11 cases of croupous pneumonia, and of these 9 recovered and 2 died; and 7 cases of acute rheumatism, all of whom recovered. The operation table does not give results. There were 10 cases of abdominal section for appendicitis, and on reference to the cause of death table, we notice two deaths from this disease, but on turning back to the general table we find there were 23 cases altogether, so we are left in doubt whether the two deaths were or were not subsequent to operation. Again, the operation table tells us there was one amputation through the hip-joint, and the death table has in it a death from lacerated thigh; but we have no means of knowing whether these entries refer to the same patient or to different patients. There is no table showing the nature of the anæsthetic used. Of the 42 deaths, 15 died within 24 hours of admission.

### DURHAM COUNTY HOSPITAL.

The gross revenue amounted to £4,005, and the gross expenditure to £2,944, showing a most favourable state of finances. The workpeople's own subscriptions amounted to £1,828, which is quite a reasonable sum to expect from the population of the district. The committee were able to place £1,500 on deposit, and had £512 in hand as a working balance at the end of the year. The information supplied on other points is extremely meagre. We notice no statement of the cost per bed occupied, the cost per patient, the cost per head per week, or the average duration of treatment. At the beginning of the year there were 45 in-patients and 631 were admitted during the year, making a total of 676. Of these 491 were discharged cured, 99 were discharged relieved, 45 died, and 41 remained in the hospital. The highest number resident on any one day was 51, and the lowest was 19; the average daily number being 38. There were 330 operations performed under anæsthetics; but no table is given showing the nature of the agents used. The medical and surgical tables are practically useless as they merely show the numbers treated for certain diseases.

### BIRMINGHAM FREE HOSPITAL FOR SICK CHILDREN.

The income was £5,252, and the expenditure £5,467, showing a deficit of £215, and increasing the deficiency in the revenue account to £700, a fact which the committee states points to the urgent necessity of new and increased subscriptions. The cost per head per week of in-patients was 2s. 11d., that of officers was 6s. 5d. On January 1st the hospital contained 55 patients, and 864 were admitted during the year, making a total of 919 under treatment. Of these, 512 were discharged cured, 209 were discharged relieved, 37 not relieved, 97 died, and 64 remained in the hospital. The average stay in the wards was 21 days. The medical and surgical tables convey no information beyond the number of cases of the various diseases named, but a table of deaths and their causes is given and by its aid it is possible to extract some information. For instance there were 75 cases of diphtheria and on referring to Table X we find there were 16 deaths from that disease, so we may imagine that 59 cases were either cured, relieved, or remained under treatment. In 24 cases of enteric fever there was only 1 death. The death-rate, reckoning all the deaths, was 11·2 per cent. The total number of operations performed was 2,300, but there is no anæsthetic table given.

